# Knowledge and confidence in managing obstructive sleep apnea patients in Canadian otolaryngology - head and neck surgery residents: a cross sectional survey

**DOI:** 10.1186/s40463-020-00417-6

**Published:** 2020-04-23

**Authors:** Saad Ansari, Amanda Hu

**Affiliations:** Division of Otolaryngology-Head and Neck Surgery, Department of Surgery, University of British Columbia, 2775 Laurel Street, 4th floor, Vancouver, BC V5Z 1M9 Canada

**Keywords:** Obstructive sleep apnea, Medical education, Resident, Otolaryngology - head and neck surgery

## Abstract

**Background:**

Obstructive sleep apnea is an expected competency for Otolaryngology - Head and Neck surgery residents and tested on the Royal College of Physicians and Surgeons examination. Our objective was to evaluate the knowledge, attitudes and confidence of Canadian Otolaryngology - Head and Neck surgery residents in managing Obstructive Sleep Apnea (OSA) patients.

**Methods:**

An anonymous, online, cross-sectional survey was distributed to all current Canadian Otolaryngology-Head and Neck surgery residents according to the Dillman Tailored Design Method in English and French. The previously validated OSA Knowledge and Attitudes (OSAKA) questionnaire was administered, along with questions exploring resident confidence levels with performing OSA surgeries. Descriptive statistics, Wilcoxon Rank Sum and unpaired Student’s t tests were calculated in Excel.

**Results:**

Sixty-six (38.4%) out of 172 residents responded (60.6% male; 80.3% English-speaking). Median OSAKA knowledge score was 16/18 (88.9%; Interquartile range: 14–16). Although all respondents believed that OSA was an important clinical disorder, only 45.5% of residents felt confident in managing OSA patients, while only 15.2% were confident in managing continuous positive airway pressure therapy (CPAP). Senior residents were more confident than junior residents in identifying OSA patients (96.7% vs 69.4%; *p* < 0.005) and managing the disease (60.0% vs. 33.3%; *p* = 0.03), including CPAP (26.7% vs. 5.6%; *p* = 0.01).

Residents had lowest confidence levels in performing tongue base suspension (1.5%), transpalatal advancement pharyngoplasty (3.0%), and laser assisted uvulopalatoplasty (6.1%). Highest confidence levels were described in performing septoplasty (56.1%), adult tonsillectomy (75.8%), and tracheotomy (77.3%).

**Conclusions:**

Otolaryngology - Head and Neck surgery residents’ knowledge of OSA was very good; however, confidence levels for managing OSA and performing OSA surgeries were varied. Several areas of perceived strengths and weaknesses in OSA training were identified by Canadian Otolaryngology - Head and Neck surgery residents.

## Background

Obstructive sleep apnea (OSA) is a highly prevalent sleep-related breathing disorder in Canada. The Canadian 2009 Sleep Apnea Rapid Response Questionnaire estimated that the prevalence rate was 3% among adults 18 years or older [1]. This statistic is not dissimilar to the 3.1–7.5% prevalence rates found in United States, Australia, India, China and Korea [2]. Canadian adults diagnosed with sleep apnea were more likely to have other health conditions, such as diabetes, hypertension, heart disease, and various mood disorders [1]. First-line treatment is a trial of nocturnal continuous positive airway pressure (CPAP) ventilation. A comprehensive literature review by Rotenberg et al. in 2016 revealed that non-adherence to CPAP was 34.1% [3]. Therefore, second line options are now becoming more prevalent. Surgical procedures for sleep apnea are being used with variable success to manage or potentially cure OSA.

Management of OSA is an expected competency for Otolaryngology - Head and Neck Surgery residents and tested on the Royal College of Physicians and Surgeons examination. Competency based education recognizes the importance of OSA as noted in Entrustable Professional Activity (EPA) #9, which states that a resident must be able to assess and manage adult and pediatric patients with sleep disordered breathing [4]. As a result, training residents to understand the nature of OSA and making them confident in performing sleep surgeries is crucial. A US-based study from Stanford by Gouveia et al. found that programs with a dedicated sleep surgeon were more likely to respond “extremely” or “very” satisfied when asked about resident exposure to sleep surgery compared to those who did not have a sleep surgeon [5].

The objective of this study was to determine the level of OSA knowledge, attitudes and confidence amongst Canadian Otolaryngology - Head and Neck Surgery residents. The study also aimed to determine the confidence levels of residents in performing various sleep apnea surgeries. Upon review of PubMed, EMBASE and Medline, no study to date has answered these questions for Canadian Otolaryngology - Head and Neck Surgery residents.

It was hypothesized that Canadian Otolaryngology - Head and Neck Surgery residents are not fully confident with managing OSA.

## Methods

### Design and study population

This study was approved by the University of British Columbia Research Ethics Board (REB # H19–2007) and consent was obtained from each participant prior to survey initiation. An anonymous, online, cross-sectional survey was sent to all current Otolaryngology-Head and Neck Surgery residents in Canada (*n* = 172), including both English and French programs, between October and November 2019. Canadian Otolaryngology - Head and Neck surgery residents enter directly into a five-year residency program after medical school. Residents perform some off-service rotations, but the majority of their time is spent practicing Otolaryngology-Head and Neck Surgery. Junior residents were defined as post-graduate years (PGY) 1, 2, and 3. Senior residents were defined as PGY 4 and 5.

### Distribution

The survey was distributed according to the Dillman Tailored Design Method. This methodology is used by the US Census Bureau and Gallup Organization and has been shown to enhance response rates [6]. Respondents were contacted multiple times over an 8-week period through email. In week 1, an invitation to participate and survey link were provided. In week 2, the first reminder was sent with the survey link. In week 4, a second reminder with the survey link and a thank you to those who had participated were distributed. In week 7, a third reminder to participate with the survey link and a thank you were sent. After week 8, survey enrollment was closed. The survey was created and hosted on the University of British Columbia’s secure Qualtrics Survey program.

### Survey structure

The survey consisted of two components. The first part incorporated the validated OSAKA (Obstructive Sleep Apnea Knowledge and Attitudes) questionnaire, which evaluates the knowledge, attitudes and confidence of the respondent on the topic of OSA [7]. OSAKA has been used in several studies worldwide and has been translated from English into Spanish and Italian [8–11]. There were three subsections within the questionnaire. The first subsection evaluated basic OSA knowledge and generated the Knowledge score. Respondents answered 18 general knowledge questions on OSA, shown in Table 1. Respondents options included “True”, “False”, and “Unsure/Do Not Know”. The second subsection delineated the attitudes of the respondent with respect to the importance of OSA, which generated the Attitude Score (two questions), shown in Table 1. In this subsection, respondents replied on a five-point scale, from 1 (Not Important) to 5 (Extremely Important). The third subsection determined the confidence levels in managing the condition and it generated the Confidence Score (three questions), shown in Table 1. In this subsection, respondents answered on a five-point Likert scale, from 1 (Strongly Disagree) to 5 (Strongly Agree).

**Table 1:** OSAKA Questionnaire Responses

OSAKA Questionnaire Responses	
Knowledge Questions	Number of Correct Responses (%)
1. Women with OSA may present with fatigue alone. (**True**)	57 (86.4%)
2. Uvulopalatopharyngoplasty is curative for the majority of patients with OSA. (**False**)	59 (89.4%)
3. The estimated prevalence of OSA among adults is between 2 and 10%. (**True**)	35 (53.0%)
4. The majority of patients with OSA snore. (**True**)	53 (80.3%)
5. OSA is associated with hypertension. (**True**)	62 (93.9%)
6. An overnight sleep study is the gold standard for diagnosing OSA. (**True**)	60 (90.9%)
7. CPAP (continuous positive airway pressure) therapy may cause nasal congestion. (**True**)	38 (57.6%)
8. Laser-assisted uvuloplasty is an appropriate treatment for severe OSA. (**False**)	27 (40.9%)
9. The loss of upper airway muscle tone during sleep contributes to OSA. (**True)**	65 (98.5%)
10. The most common cause of OSA in children is the presence of large tonsils and adenoids. (**True**)	66 (100%)
11. A craniofacial and oropharyngeal examination is useful in the assessment of patients with suspected OSA. (**True**)	65 (98.5%)
12. Alcohol at bedtime improves OSA. (**False**)	63 (95.5%)
13. Untreated OSA is associated with a higher incidence of automobile crashes. (**True**)	61 (92.4%)
14. In men, a collar size 17 in. or greater is associated with OSA. (**True**)	53 (80.3%)
15. OSA is more common in women than in men. (**False**)	64 (97.0%)
16. CPAP is the first line therapy for severe OSA. (**True**)	60 (90.9%)
17. Less than 5 apneas or hypopneas per hours is normal in adults. (**True**)	56 (84.8%)
18. Cardiac arrhythmias may be associated with untreated OSA. (**True**)	60 (90.9%)
**Median Total Knowledge Score (IQR)**	**16 (14–16)**
**Attitude Questions** Not important = 1, Somewhat important = 2, Important = 3, Very important = 4, Extremely important = 5	Median Score out of 5 (Interquartile Range)
1. As a clinical disorder, OSA is:	4(3–4)
2. Identifying patients with possible OSA is:	4(3–4)
**Median Total Attitude Score**	**4(3–4)**
**Confidence Questions** Strongly disagree = 1, Disagree = 2, Neither agree nor disagree = 3, Agree = 4, Strongly Agree = 5	Median out of 5 (Interquartile Range)
1. I feel confident identifying patients at-risk for OSA:	4(4–4)
2. I am confident in my ability to manage patients with OSA:	3(3–4)
3. I am confident in my ability to manage patients on CPAP therapy:	3(2–3)
**Mean Total Confidence Score**	**3(3–4)**

The second component to the survey was a confidence questionnaire for various procedures involved in the surgical management of OSA from the perspective of the Otolaryngologist - Head and Neck Surgeon. This component was an addition created by the authors to target the survey to a surgical audience. This component included sixteen different procedures that have been proven in the literature to treat OSA (Table 2). The respondents answered on a five-point Likert scale, from 1 (Strongly Disagree) to 5 (Strongly Agree). The five-point Likert scale was used in the OSAKA questionnaire and in previously published studies on medical education, for example, in a survey of laryngology education among Canadian otolaryngology residents [12] and otolaryngology education among Canadian family medicine residents [13]. Demographic data, including level of training, language of training, and gender of the respondents were also collected. Level and language of training data were collected to compare the results among junior versus senior residents and English and French training programs.

**Table 2:** Resident Confidence Scores in performing OSA procedures, in descending order of confidence levels

Confidence in Performing OSA Procedures	
Procedure	Confidence Level Median (Interquartile Range) *Strongly disagree = 1, Disagree = 2, Neither agree nor disagree = 3, Agree = 4, Strongly Agree = 5*
1. Tracheotomy	5(4–5)
2. Tonsillectomy	5(4–5)
3. Septoplasty	4(2–5)
4. Uvulopalatopharyngoplasty (UPPP)	2.5(2–4)
5. Lingual Tonsillectomy	3(2–4)
6. Septorhinoplasty	2(2–4)
7. Drug-Induced Sleep Endoscopy (DISE)	2(1.25–3.75)
8. Radiofrequency Ablation of Base of Tongue (RFBOT)	2(1–3.75)
9. Lateral Pharyngoplasty	2(1–3)
10. Midline Glossectomy	2(1–3)
11. Radiofrequency Ablation of Soft Palate (RFSP)	2(1–3)
12. Expansion Sphincter Pharyngoplasty	2(1–2)
13. Laser Assisted Uvuloplasty (LAUP)	2(1–2)
14. Base of Tongue (BOT) Suspension	2(1–2)
15. Palate Implants	1(1–2)
16. Transpalatal Advancement Pharyngoplasty (TAP)	1(1–2)
**Median Total Confidence Score**	**2(1–4)**

### Statistical analysis

The knowledge, attitude and confidence scores as part of the OSAKA Questionnaire, along with the confidence scores in the Otolaryngology-Head and Neck surgery procedure section, were all calculated as median, interquartile ranges (IQR), and percentages. The results were compared between levels of training (Junior – PGY1–3 vs. Senior – PGY4–5), language of training (English vs. French) and Gender (Male vs. Female) using either a Wilcoxon Rank Sum test or a Students unpaired t-test. Statistical calculations were performed on Microsoft Excel and an a priori probability level of *p* < 0.05 was considered to be significant.

## Results

Sixty-six responses were received from 172 residents across Canada for a response rate of 38.4%. Male respondents comprised 60.6% and 80.3% were English-speaking (Table 3).

**Table 3:** Demographic Survey Data

Baseline Characteristics	N (%)
**Total Respondents**	**66 (38.4% Response Rate)**
**Gender**	
Male	40 (60.6%)
Female	26 (39.4%)
**Language of Training**	
English	53 (80.3%)
French	13 (19.7%)
**Level of Training**	
Post Grad Year 1	13 (19.7%)
Post Grad Year 2	15 (22.7%)
Post Grad Year 3	8 (12.1%)
**Total Juniors (PGY1–3)**	**36 (54.5%)**
Post Grad Year 4	17 (25.8%)
Post Grad Year 5	13 (19.7%)
**Total Seniors (PGY 4–5)**	**30 (45.5%)**

### OSAKA knowledge scores

Median knowledge score for all respondents was 16 out of 18 (88.9%) with an IQR between 14 and 16.. For each question, the percentage of respondents who answered correctly are noted in Table 1.As expected, junior residents’ median knowledge scores were significantly lower (83.3%, IQR 76.4–88.9%) compared to senior residents’ (88.9%, IQR 83.3–94.4%; *p* = 0.0219). There were no statistically significant differences between gender or language of training. The three questions with the lowest scores on the questionnaire were related to laser-assisted uvuloplasty not being a treatment for severe OSA (40.9%), the prevalence of OSA (53.0%), and CPAP causing nasal congestion (57.6%). The rest of the knowledge questions were answered well with scores above 80%.

### OSAKA attitude scores

The median two-question attitude score for all respondents was 4 out of 5 (IQR, 3–4) as shown in Table 1. Overall, all respondents considered OSA to be an important clinical disorder. All respondents reported that it was important to be able to identify patients who are at risk of OSA.

### OSAKA confidence scores

The median three-question confidence score for all respondents was 3 out of 5 (IQR, 3–4), as shown in Table 1. There were no statistically significant differences based on gender and language of training.

**Fig. 1:**
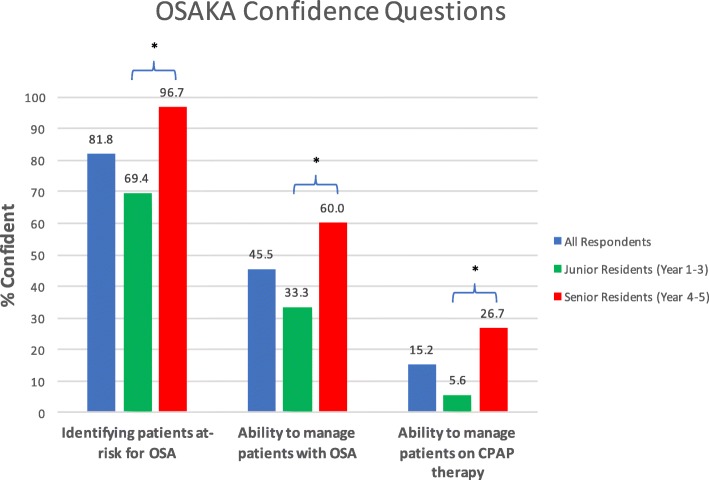
*Confidence Questions Responses*. The percentage of respondents who answered “Agree” or “Strongly Agree” to the Confidence-related questions on the OSAKA Questionnaire. Responses are shown as all respondents (blue), junior residents (post graduate years 1–3) (green), and senior residents (post graduate years 4–5) (red). *Significant *p* < 0.05

When reviewing each of the three confidence questions separately, 81.8% of respondents “agreed” or “strongly agreed” that they were confident in identifying patients with OSA (Fig. 1). However, only 45.5 and 15.2% of respondents “agreed” or “strongly agreed” that they were confident in managing OSA patients and CPAP therapy, respectively. The proportion of Seniors who answered either “agree” or “strongly agree” was significantly higher than Juniors in identifying OSA (96.7% vs 69.4%; *p* < 0.005), managing OSA (60.0% vs. 33.3%; *p* = 0.03) and managing CPAP (26.7% vs. 5.6%; *p* = 0.01) confidence questions. Despite this improvement over the Junior residents, senior confidence levels in management of OSA and CPAP were still low (60.0 and 26.7%, respectively). No significant differences were found between gender and language of training.

### Surgical procedures for OSA

**Fig. 2:**
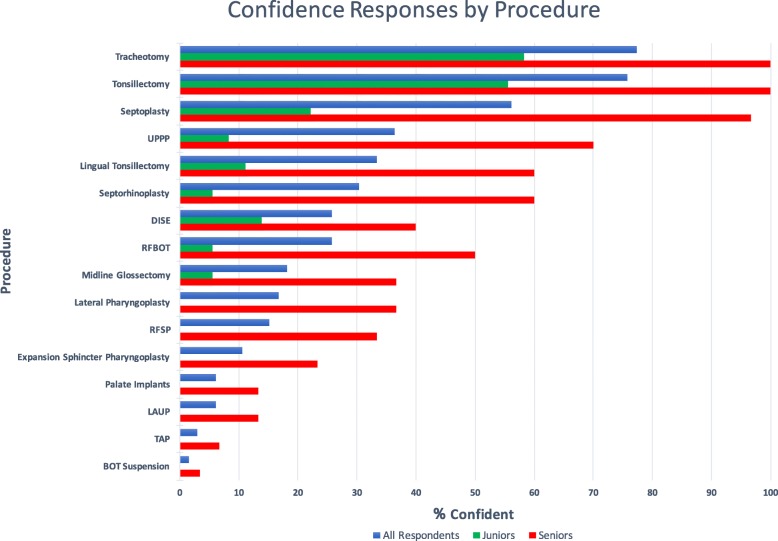
Percentage of Respondents who answered “Agree” or “Strongly Agree” for each OSA-related surgical procedure, in descending order of confidence levels. Responses are shown for all respondents (blue), junior residents (post graduate years 1–3) (green), and senior residents (post grduate years 4–5) (red). Abbreviations: BOT, Base of Tongue; RFBOT, Radiofrequency Ablation of Base of Tongue; RFSP, Radiofrequency Ablation of Soft Palate; TAP, Transpalatal Advancement Pharyngoplasty; LAUP, Laser Assisted Uvulopalatoplasty; UPPP, Uvulopalatopharyngoplasty; DISE, Drug-Induced Sleep Endoscopy

Confidence levels in performing OSA-related surgeries were analyzed on a five-point scale, where 1 is strongly disagree and 5 is strongly agree. OSA-related surgeries are listed in decreasing order of confidence levels in Table 2. Overall, the top three procedures that had an “agree” or “strongly agree” response were tracheotomy (77.3%), tonsillectomy (75.8%), and septoplasty (56.1%). The procedures with the lowest three responses were tongue base suspension (1.5%), transpalatal advancement pharyngoplasty (3.0%) and laser assisted uvulopalatoplasty (6.1%). Fig. 2 displays the results for all respondents, junior residents (PGY 1–3) and senior residents (PGY 4–5), in decreasing order of confidence levels.

## Discussion

This was the first study to use the validated OSAKA questionnaire to determine the knowledge, attitudes and confidence levels of Canadian Otolaryngology - Head and Neck Surgery residents. A thorough review of the literature revealed five other studies using the OSAKA questionnaire, albeit in different study populations [7–11]. A head to head comparison of those studies to the current study may not be perfect, however, salient themes are identified by reviewing the methodology and results of these studies.

The OSAKA questionnaire was pilot tested by 20 internists who were attending a hospital sponsored lecture on OSA. The questionnaire was then mailed out and validated in a group of 115 physicians who were internists, pediatricians or family practitioners associated with the Washington University Physicians Network [7]. Overall, the mean knowledge score was 13.3 ± 2.8 (73.9%), and scores did not differ significantly by gender or specialty training. Knowledge score was negatively correlated with age and confidence level; thus, older respondents had lower knowledge scores and were less confident with managing OSA patients.

In the second study, the OSAKA questionnaire was used in a cross-sectional survey of graduating medical students in Nigeria [8]. This study had the highest response rate of 99%, generating a sample size of 143. The mean OSAKA knowledge score was 7.6 ± 3.2 (42.2%), and scores did not differ significantly with age or gender. This study concluded that sleep disorders should be formally incorporated into the undergraduate medical curriculum in Nigeria.

In the third study, the OSAKA questionnaire was translated into Italian and distributed to anesthetists attending a national conference [9]. In total, 370 anesthetists (62% response rate) scored a median of 12 (interquartile range 10–14) with no significant difference by gender, age, professional title (resident vs attending), and years in practice. For the attitude score, females and anesthetists with > 15 years of practice scored higher, while residents scored lower.

In the fourth study, the OSAKA questionnaire was translated into Spanish and distributed to primary care physicians attendings medical conferences in six Latin American cities: Quito and Guayaquil, Lima, Peru, and Caracas, Valencia, and Maracaibo, Venequela [11]. In total, 367 participants (65% response rate) scored a mean of 60% and this did not significantly differ due to age or year of graduation.

In the last study, the authors modified the OSAKA questionnaire to evaluate American cardiologists [10]. The authors added 20 questions on the relationship between sleep apnea and cardiovascular diseases. In total, 92 cardiologists (22% response rate) had a mean knowledge score of 76%. Scores were not significantly different based on age, gender, subspecialty, or years of practice. Our study followed the precedence set by this paper’s methodology by adding items to the OSAKA questionnaire to adapt to a different specialty audience.

Our study was different than the previous studies in several ways. Our study population was Otolaryngology – Head & Neck Surgery, the first surgical specialty to use the OSAKA questionnaire. Many different specialties treat OSA patients; thus, many specialties are interested in OSA education. Previous studies surveyed physicians at various professional levels, ranging from medical students to attendings. Our study focused solely on residents as we were interested in resident education. Lastly, the methodology of online survey administration was different. This change may reflect the adoption of new technology. The questionnaire was first validated in 2008 with a mail out survey methodology. Subsequent surveys were distributed at conferences or during medical school. In 2020, online surveys are commonly used and was the methodology employed by the current study.

Our response rate was 38.4%, which is around the accepted average of online surveys (34%) [14]. Our response rate was also similar to other surveys on resident education in otolaryngology. A previous study of Canadian otolaryngology residents on laryngology education had a response rate of 42.6% [12]. Two previous studies of American otolaryngology chief residents on rhinology and pediatric education had response rates of 26 and 17.6% respectively [15, 16]. Our response rate was also between the range reported by the five other studies that used the OSAKA questionnaire (i.e. 22 to 99%) [7–11].

In terms of knowledge, residents in this specialty were considered to be quite insightful into the disease as the median OSAKA knowledge score was 88.9%. This questionnaire was implemented in a variety of other specialties at various levels of training ranging from medical student to attending [7–11]. The mean knowledge scores from these previous studies were all lower than the median knowledge score in the current study, ranging from 42.2 to 77%. Therefore, the results from our study show that, as experts of the upper airway, Otolaryngology - Head and Neck surgery residents have an all-round grasp on the basic knowledge concepts of OSA.

In terms of attitude towards managing patients with OSA, there was an overwhelming consensus amongst the residents that it is an important skill to acquire for the specialty. The high level of importance noted by the respondents could be explained by the fact that procedures in Otolaryngology - Head and Neck surgery frequently require manipulation of the upper airway, which can be adversely affected by the presence of OSA.

In the final section of the original OSAKA questionnaire, the goal was to evaluate the confidence levels in diagnosing and managing these patients from the perspective of the Otolaryngologist - Head and Neck surgeon. Specifically, the resident respondents were comfortable in identifying the patients who have OSA (81.8%). However, managing these patients was considerably lower with only 45.5% of respondents being confident. This confidence level increased only to 60% in the senior resident group (*p* = 0.03). Therefore, confidence was not appropriately developed when residents advance through Canadian Otolaryngology - Head and Neck surgery training programs.

These lower confidence levels are in contrast to the high knowledge levels noted in this study. Residents have OSA knowledge, but do not feel confident in managing patients with the condition. There may be limited or missed opportunities in residency programs to manage OSA patients who are surgical candidates. This may be due to the infancy of the subspecialty of sleep medicine and/or the lack of fellowship trained sleep surgeons in academic otolaryngology training programs. A survey of residency program directors in the United States [5] showed that only 41.3% of Otolaryngology - Head and Neck surgery programs have faculty who dedicate more than 50% of their time to sleep surgery. Approximately 70% of respondents in that survey study also agreed or strongly agreed that there would be a benefit for residents in having a sub-specialty trained sleep surgeon on faculty. It is very likely that this same result would be applicable to Canadian training programs. However, further research is needed to ascertain the reasons for the lack of confidence noted in this study.

With respect to CPAP management, it is understandable that respondents were the least confident in this subject area among the three confidence questions (15.2%). In most cases, respirologists and not otolaryngologist - head and neck surgeons, are managing and prescribing CPAP. Despite this, the lack of confidence in how to manage CPAP therapy can hinder the ability of the otolaryngologist - head and neck surgeon to provide comprehensive care for OSA patients.

Several factors were evaluated to determine if they had an impact on OSAKA scores and confidence levels. These factors were explored based on the previous literature. A cross-sectional survey of Canadian Otolaryngology- Head & Neck Surgery residents was conducted on laryngology education in 2012. Variables that were explored included level of training (junior vs senior), language of instruction (English vs French), presence of a fellowship, completion of an elective or research project in laryngology, age, and gender. This study reported that the only factor that was significant was level of training. Overall, senior residents were more comfortable than junior residents with providing laryngology care [12]. Our current study confirms these findings. For the validated OSAKA confidence items shown in Fig. 1, senior residents were more confident than junior residents in identifying OSA patients (96.7% vs 69.4%; *p* < 0.005) and managing the disease (60.0% vs. 33.3%; *p* = 0.03), including CPAP (26.7% vs. 5.6%; *p* = 0.01). For the confidence levels by surgical procedure shown in Fig. 2, senior residents had higher confidence levels for every surgery as compared to junior residents. These findings make intuitive sense.

The five previous OSAKA studies also explored several variables: gender, age, year of practice, time since graduation, professional title (attending vs resident) [7–11]. Overall, none of these factors had a significant impact on the knowledge scores. Only one previous study had an interesting result. The study on Italian anesthetists reported a significant difference in the altitude scores [9]. Females and anaesthetists with > 15 years of practice reached higher attitude scores, while anaesthesia residents showed a lower attitude score. Our current study reported that language of instruction and gender did not have any substantial effect on knowledge, attitudes or confidence responses of the residents. These factors were not hypothesized to be significant prior to analyzing the results of the study.

In the additional component of the survey, the confidence levels of the respondents in performing OSA surgeries were recorded. Not surprisingly, the highest confidence levels were recorded for procedures that are performed not only for OSA, but also for other clinical indications. These include tracheotomy (77.3%), tonsillectomy (75.8%) and septoplasty (56.1%). However, procedures that are more specific to OSA, including DISE, UPPP, palatal and tongue base procedures, had lower confidence levels. According to the recently introduced competency by design curriculum for Otolaryngology - Head and Neck Surgery implemented by the Royal College of Physicians and Surgeons of Canada, the Entrustable Professional Activities (EPA) Guide has outlined in core EPA#9 that residents must be able to manage a patient with sleep-disordered breathing and also perform at least one palate procedure [4].

There are some limitations to this study. Firstly, we used the OSAKA sleep questionnaire which is a validated questionnaire for physicians’ knowledge and attitudes [7]. However, the questions on level of confidence for sleep surgery procedures were not validated. We attempted to adapt the OSAKA questionnaire, which was originally validated in non-surgeons, to a surgical audience. The use of an ad-hoc assessment tool limits generalizability to other groups or populations. In our thorough literature search, we could not find another validated questionnaire that was more suitable for our present study. Secondly, we are limited by the response rate of 38.4%. Although this response rate is similar to other studies [12, 15, 16], there may be a selection bias among the residents who chose to answer the survey. Administration of an anonymous survey is subject to numerous biases including design, ascertainment and selection bias, limiting the observations to sampled population only. Lastly, these data focus on knowledge, attitudes and confidence of Otolaryngology-Head and Neck Surgery residents from their perspective. It does not provide information on number of sleep procedures performed or number of EPAs completed. It is also not an audit of the sleep medicine curriculum in Canadian Otolaryngology – head and neck surgery residency programs. Confidence of residents may not necessarily translate into competence.

## Conclusion

Otolaryngology - Head and Neck Surgery residents reported that knowledge and understanding of OSA is important in their specialty. Their confidence, however, in managing this condition and performing OSA surgeries was varied, despite achieving very high scores on the basic OSA knowledge testing. Since obstructive sleep apnea is an expected competency by the Royal College of Physicians & Surgeons of Canada, training in this area is crucial. Areas of perceived strengths and weaknesses have been explored in this study by the respondents, who are key stakeholders in this educational process. This study was the first survey of OSA knowledge, attitudes, and confidence levels in Canadian Otolaryngology - Head & Neck Surgery residents.

## Data Availability

The datasets used and/or analysed during the current study are available from the corresponding author on reasonable request.

## Abbreviations

OSA: Obstructive Sleep Apnea
CPAP: Continuous Positive Airway Pressure
EPA: Entrustable Professional Activity
OSAKA: Obstructive Sleep Apnea Knowledge and Attitudes
IQR: Interquartile Range
UPPP: Uvulopalatopharyngoplasty
DISE: Drug-Induced Sleep Endoscopy
RFBOT: Radiofrequency Ablation of Base of Tongue
RFSP: Radiofrequency Ablation of Soft Palate
LAUP: Laser-assisted Uvuloplasty
TAP: Transpalatal Advancement Pharyngoplasty
BOT: Base of Tongue
